# Broadening horizons: intestinal microbiota as a novel biomarker and potential treatment for hypertensive disorders of pregnancy

**DOI:** 10.3389/fcimb.2024.1446580

**Published:** 2024-08-22

**Authors:** Min Wang, Lianwen Zheng, Yang Meng, Shuai Ma, Donghai Zhao, Ying Xu

**Affiliations:** ^1^ Department of Obstetrics and Gynecology, The Second Hospital of Jilin University, Changchun, China; ^2^ Jilin Province Product Quality Supervision and Inspection Institute, Changchun, China; ^3^ Department of Pathology, Jilin Medical College, Jilin, China

**Keywords:** intestinal microbiota, gut flora, hypertensive disorders of pregnancy, metabolism disorder, treatment, expression, biomarkers

## Abstract

Hypertensive disorders of pregnancy (HDP) are severe complications of pregnancy with high morbidity and are a major cause of increased maternal and infant morbidity and mortality. Currently, there is a lack of effective early diagnostic indicators and safe and effective preventive strategies for HDP in clinical practice, except for monitoring maternal blood pressure levels, the degree of proteinuria, organ involvement and fetal conditions. The intestinal microbiota consists of the gut flora and intestinal environment, which is the largest microecosystem of the human body and participates in material and energy metabolism, gene expression regulation, immunity regulation, and other functions. During pregnancy, due to changes in hormone levels and altered immune function, the intestinal microecological balance is affected, triggering HDP. A dysregulated intestinal microenvironment influences the composition and distribution of the gut flora and changes the intestinal barrier, driving beneficial or harmful bacterial metabolites and inflammatory responses to participate in the development of HDP and promote its malignant development. When the gut flora is dysbiotic and affects blood pressure, supplementation with probiotics and dietary fiber can be used to intervene. In this review, the interaction between the intestinal microbiota and HDP was investigated to explore the feasibility of the gut flora as a novel biomarker of HDP and to provide a new strategy and basis for the prevention and treatment of clinical HDP.

## 1 Introduction

Hypertensive disorders of pregnancy (HDP) and its complications are direct causative factors of maternal and infant mortality (Hu et al., 2019). Due to the special physiological and metabolic conditions of pregnant women, clinical diagnosis and therapeutic interventions for HDP are limited. A large number of intestinal microbes significantly impacts human health and disease, and its genome is known as the “second genome” of humans (Marchesi et al., 2016). The intestinal microbiota plays an essential role in human health and disease through interactions with the host intestinal environment (Rosenbaum et al., 2015; Ron et al., 2016). Research related to the intestinal microbiota has shown great potential clinical value in various diseases, such as heart failure, atherosclerosis, and neurological diseases (Hotamisligil, 2006). With the development of next-generation sequencing technology, research in the field of intestinal microbiota has become a hotspot in basic, clinical, and translational medicine (Jin et al., 2019). The gut flora and human genome influence the host’s nutrition, metabolism, immunity, behavior, stress, and many other physiological processes through interactions with the external environment (Marques et al., 2017). The gut flora and its metabolites play essential roles in the development of metabolic diseases, such as HDP, during pregnancy (Angelakis et al., 2012). Dysbiosis of the gut flora during HDP reflects the disease status of pregnant women and can be used as a clinical diagnostic marker (Yoo et al., 2016). It is possible to monitor maternal gut flora as a routine clinical diagnostic strategy to help with early detection, auxiliary diagnosis, clinical treatment, and disease prediction of HDP (Mushtaq et al., 2019). This article reviews the association between the intestinal microbiota and HDP, analyses the current research status of HDP, the application value of the gut flora and its metabolite-related indicators, and explores the feasibility of the gut flora as a novel marker of HDP.

## 2 HDP overview

**Figure 1:**
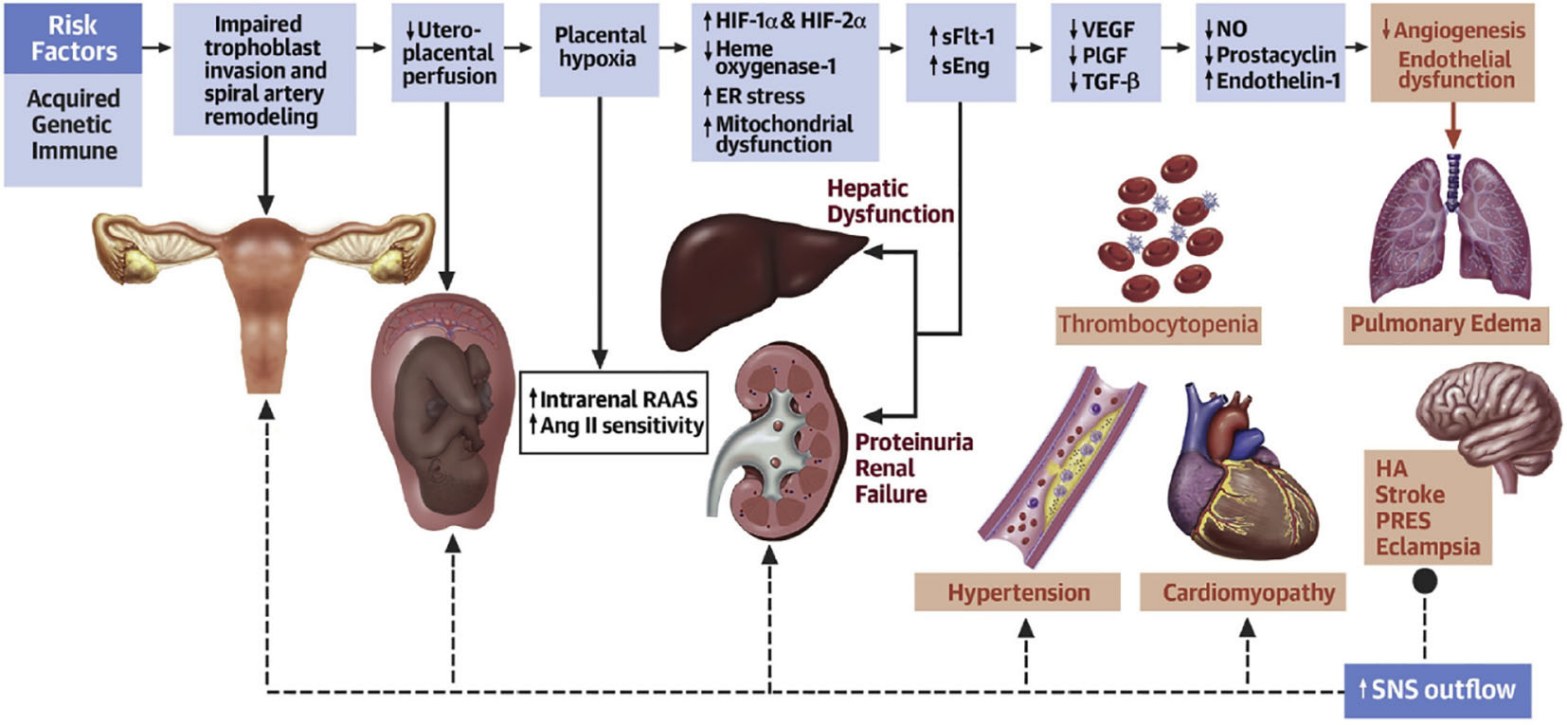
Acquired, genetic, and immune risk factors contribute to early placental dysfunction. Placental dysfunction results in the release of antiangiogenic factors, leading to subsequent multiorgan dysfunction. The solid arrows represent the progression of disease. The dashed arrows represent the effects of SNSs on the respective organs. Ang II, angiotensin II; ER, endoplasmic reticulum; HA, headache; HIF, hypoxia-inducible transcription factor; NO, nitric oxide; PlGF, placental growth factor; PRES, posterior reversible encephalopathy syndrome; RAAS, renin-angiotensin-aldosterone system; sEng, soluble endoglin; sFlt, soluble fms-like tyrosine kinase; SNS, sympathetic nervous system; TGF, transforming growth factor; VEGF, vascular endothelial growth factor (Ives et al., 2020).

HDP is population specific, most cases are temporary hypertension, which returns to normal after delivery, and approximately one-fourth of patients progress, with symptoms such as proteinuria, edema, and, in severe cases, convulsions, coma, and multiple organ involvement (Mosimann et al., 2020). HDP usually appears at approximately 20 weeks of gestation and is clinically categorized as gestational hypertension, preeclampsia (PE), or eclampsia based on severity (Ives et al., 2020) (**Figure 1**). Gestational eclampsia develops from the exacerbation of preeclamptic signs and symptoms. Pregnant women who are initially diagnosed with HDP are generally treated clinically conservatively with blood pressure adjustment (Nguyen et al., 2019). However, due to the large individual differences between pregnant women, congenital genetics, living environment, and dietary habits affect maternal tolerance to drugs and disease prognosis (Shen et al., 2021). The clinical management of patients with severe HDP at >28 weeks of gestation is complicated, and it is often difficult for conservative drug therapy to achieve the desired results. Overacting the inflammatory response during pregnancy, dysregulation of maternal-fetal immune homeostasis, genetic susceptibility, and individual dietary habits are closely related to HDP (Jia et al., 2018). Defining the role of intestinal dysbiosis in the occurrence and development of HDP, as well as its cause and effect, will be highly important for optimizing the clinical diagnosis and treatment of HDP (Richards et al., 2022).

## 3 Composition of the intestinal microbiota

**Figure 2:**
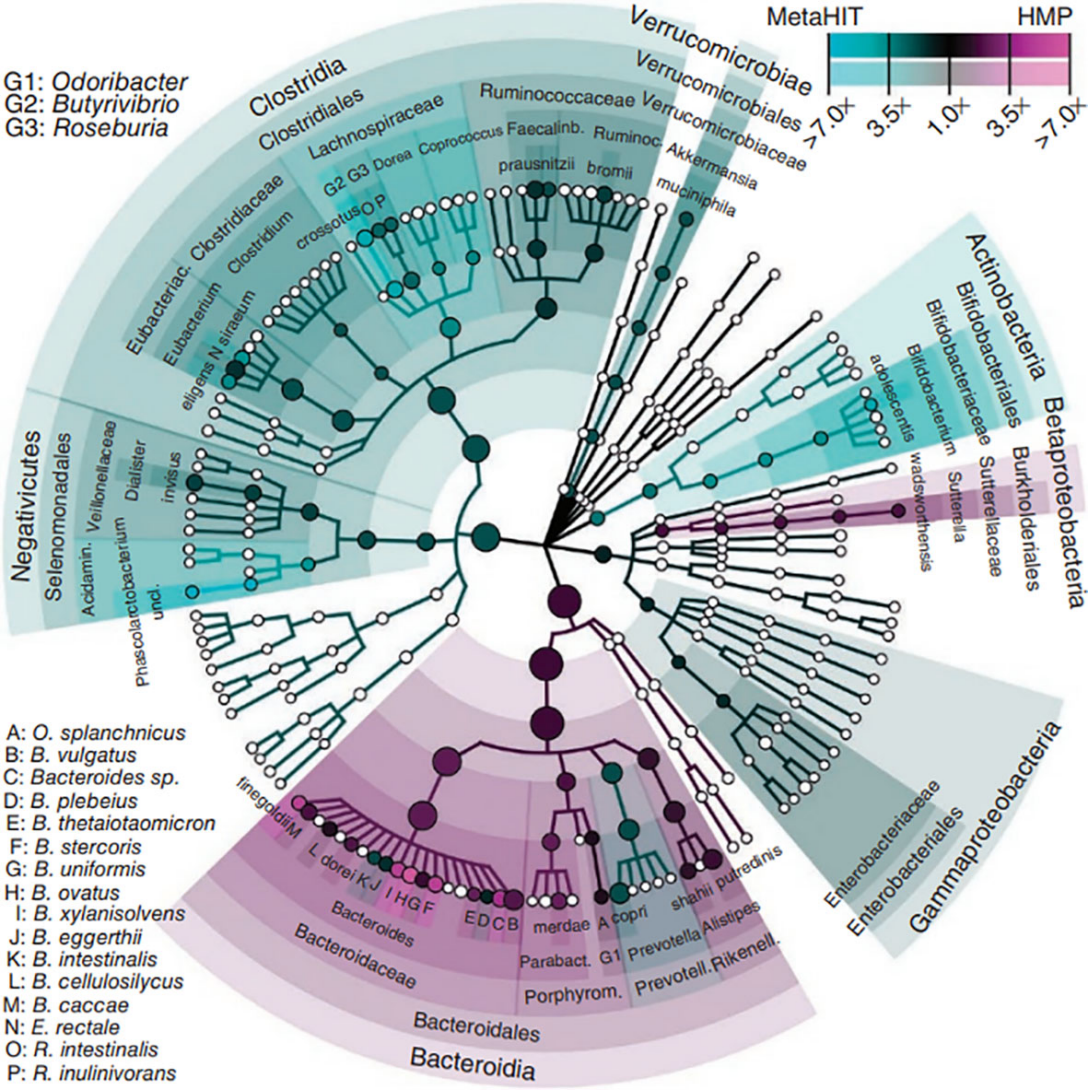
Phytogenetic tree representing the human gut microbiota. Taxonomic cladogram reporting all clades present in one or both cohorts. Circle size is proportional to the log of average abundance (Segata et al., 2012).

The intestinal microbiota refers to the gut flora and the environment in which they live and is considered the body’s largest invisible “organ” (Ma and Li, 2018). The gut is a multilayered structure, with the outer layer consisting of a mucus layer, defense proteins, an intermediate layer of intestinal epithelial cells, and an inner layer of immune cells (Cani, 2018). The intestinal microbiota are located in the outer layer of the gut and form the intestinal barrier with other intestinal structures. Intestinal immune cells and defense proteins work together to maintain intestinal immunity (Griffin, 2015). Short-chain fatty acids (SCFAs) produced by the gut flora through the metabolism of dietary fibers can regulate the mucus layer, which maintains the intestinal microbiota (Holmes et al., 2008). The gut microbial composition varies among individuals and is influenced by factors such as the environment in which the individual lives, diet, age, and genes (Callaway et al., 2016a). In healthy individuals, the intestinal microbiota is relatively stable, and healthy intestinal microbiota are characterized by diversity, stability, and tolerance and resistance and resilience to environmental changes (Noce et al., 2019). Dysregulation of the intestinal microbiota can lead to disease, and the occurrence of disease can lead to dysregulation of the intestinal microbiota (Liu et al., 2019; Wermers, 2020). Enhancing the study of the intestinal microbiota is important for promoting the management of human health (Pevsner-Fischer et al., 2017). The gut flora is the core of the intestinal microbiota (Segata et al., 2012) (**Figure 2**).

### 3.1 The gut flora

The human gut contains 1000 to 1150 species of bacteria, with at least 160 dominant species (Turnbaugh et al., 2009). The human gut flora has a symbiotic relationship with the human host (Jose and Raj, 2015). Ninety-nine percent of the bacteria belonged to five major phyla: (1) Firmicutes, such as Clostridium, Lactococcus, and Ruminococcus. (2) Bacteroidetes, such as Bacteroides, Prevotella, and other gram-negative genera. (3) Actinobacteria, such as Actinomyces, Bifidobacterium, Mycobacterium, and other gram-positive bacteria. (4) Proteobacteria, such as Salmonella, Helicobacter, Enterobacter, and other gram-negative bacteria. (5) Verrucomicrobia. Verrucomicrobia and Bacteroidetes belong to the core microbiome, which accounts for approximately 80-90% of the entire gut microflora (Eckburg et al., 2005; Lezutekong et al., 2018; Gill et al., 5778). The gut flora is divided into three categories: (1) the dominant gut flora that lives in symbiosis with humans, generally physiological anaerobes, which are the most dominant and abundant flora permanently colonizing the intestinal tract; (2) conditionally pathogenic bacteria that are predominantly parthenogenetic anaerobes and live in symbiosis with humans, which are the nondominant flora of the intestinal tract; and (3) a group of potentially pathogenic bacteria or nonpathogenic pathogens from the surrounding environment or from the host (Ley et al., 2006). The human intestinal microbiota is involved in the body’s energy storage, material transformation, mucosal barrier, and immune regulation and has an essential impact on human health and disease (Mishra et al., 2016; Yang et al., 2019).

### 3.2 Intestinal microbiota balance and dysbiosis

Although the intestinal microbiota is susceptible to some disease states, antibiotics, and dietary changes, it is in dynamic balance within certain limits (Hu et al., 2016). The intestinal microbiota balance is closely related to the immune system and inflammatory response (Qiulong et al., 2017). Overgrowth of harmful bacteria, such as enterococci and *Escherichia coli*, can disrupt the intestinal mucosal barrier and allow passage of pathogenic bacteria, activating the immune system and triggering an inflammatory response (Winter et al., 2018). Overactivity of these immune cells and inflammatory mediators can impact the blood pressure regulatory system, leading to elevated blood pressure (Adnan et al., 2017). SCFAs attenuate the hypertensive immune-inflammatory response by inhibiting the expression of CD4+ T cells, CD8+ T cells, and Th17 cells in hypertensive mice (Bartolomaeus et al., 2018). When the dynamic balance between the host and normal or harmful flora is disrupted, the harmful flora quickly grow and multiply in large quantities, resulting in a pathological state (Honour et al., 1985; Khalesi et al., 2014). Gut ecological dysregulation can cause the underproduction of vasodilators and the overproduction of vasoconstrictors through oxidized low-density lipoprotein (ox-LDL), which can lead to the development of hypertension (Kimberley et al., 2017; Gentile and Weir, 2018; Kanbay et al., 2018). Gut microecological dysregulation plays a nonnegligible role in the development of metabolic diseases during pregnancy.

### 3.3 Intestinal microbiota and hypertension

#### 3.3.1 Effect of the gut flora on hypertension

Changes in the gut flora can affect blood pressure (Lim et al., 2012). The use of multiple antibiotics to remove the intestinal microbiota from test dogs revealed a significant inhibitory effect of the test drugs on blood pressure, demonstrating the relationship between the gut flora and hypertension (Wakabayashi and Yamada, 1972). Intestinal microbiota studies using broad-spectrum antibiotics in an animal model of hypertension revealed that the treated group showed significant blood pressure decreases, demonstrating improvements in the gut environment and kidney damage (Galla et al., 2018). Broad-spectrum antibiotic elimination in patients with drug-resistant hypertension significantly decreased blood pressure in the treated patients (Qi et al., 2015). The direct effect of the intestinal microbiota on host blood pressure was demonstrated by fecal transplantation from hypertensive patients to germ-free mice, where it was observed that elevated blood pressure could be transferred through the intestinal microbiota (Li et al., 2017).

Hypertension also affects the gut flora (Katsimichas et al., 2019). Hypertension was induced in test rats using AngII, which revealed a dysregulation of the bacterial flora structure in the rat gut (Wilck et al., 2017). The microbiome of the mouse intestine is significantly altered by high salt induction, especially that of L. murinus, which was found to influence the development of hypertension by regulating TH17 cell production in the immune response mechanism (Ferguson et al., 2019). The intestinal tract of animals suffering from hypertension develops pathologic alterations manifested by decreased tight junctions, weakened mechanical barrier function, and an increased inflammatory state (Hu et al., 2017; Robles-Vera et al., 2020b). YANG et al. reported significant differences in the structure of the gut flora in the hypertensive group, with a significant decrease in the abundance and diversity of the flora, a significant decrease in beneficial bacteria, and a decrease in the number of actinomycetes and bifidobacteria compared to those in the healthy control group (Yang et al., 2015).

#### 3.3.2 Effect of gut flora metabolites on blood pressure

The metabolites of the gut microbiota include SCFAs, trimethylamine oxide (TMAO), and bile acids (Pluznick et al., 2013; Koeth et al., 2015). SCFAs include formic, acetic, propionic, butyric, isobutyric, valeric, isovaleric, and hexanoic acids (Sun et al., 2016). Approximately 90-95% of SCFAs in the colon consist of acetate, propionate, and butyrate (Puddu et al., 2014; Miyamoto et al., 2016; Vieira et al., 2017). SCFAs are produced from undigested carbohydrates, such as dietary fiber, by fermentation of the gut flora (Chen et al., 2017; Xu et al., 2022). SCFAs exert their blood pressure-regulating effects through immunomodulation, receptor action, histone deacetylase (HDAC) inhibition, and vagal stimulation to improve vascular function and attenuate the pathological process of hypertension (Vinolo et al., 2011; Den Besten et al., 2015). SCFAs mediate blood pressure regulatory mechanisms through Takeda G-protein-coupled receptor (TGR) signaling (Sean and Tara, 2017). SCFAs, especially butyrate, act as inhibitors of HDACs and mediate the downregulation of the expression of the proinflammatory phenotype of hypertension by inhibiting HDACs (Ohira et al., 2013). Animal studies have shown that the inflammatory response in spontaneously hypertensive rats is modulated by HDACs (Cardinale et al., 2010). The hypotensive effect of butyrate was attenuated in the subdiaphragmatic vagal dissociation group compared with the sham-operated group, and the hypotensive effect of butyrate also involved a reduction in sympathetic activity, suggesting that vagal signaling produced by butyrate may further intervene in hypertension by inhibiting sympathetic activity through the gut–brain axis (Onyszkiewicz et al., 2019)The gut flora metabolizes choline and phosphatidylcholine in chowder to trimethylamine via trimethylamine lyase, which is then oxidized by hepcidin monooxygenase in the liver to form TMAO through the portal system and is ultimately released into the bloodstream (Devaraj et al., 2013). TMAO can affect the concentration of cholesterol and lipoproteins in plasma, affecting blood pressure in the long term (Zhen et al., 2023). Some studies have demonstrated that the combined infusion of low-dose angiotensin II (AngII) receptor antagonist and TMAO in rats has a more pronounced antihypertensive effect and a longer duration of hypertension. The mechanism of the antihypertensive effect may be that TMAO accelerates protein folding and ligand binding, which affects the binding of specific receptors to peptide hormones, similar to Ang II, thus playing an antihypertensive role (Wang et al., 2011; SJA et al., 2021). An increase in TMAO concentration causes an increase in plasma osmolality, which induces the release of vasopressin, thereby increasing the expression of aquaporin 2 (AQP-2) and increasing the movement of AQP-2-containing vesicles to the surface of the cell membrane (Min et al., 2018). Increased expression of AQP-2 in the cell membrane leads to increased cellular permeability to water, enhanced water reabsorption, and increased blood volume, resulting in elevated blood pressure.

The intestinal microbiota regulates blood pressure by influencing steroid hormone levels (Yan et al., 2020). A high-salt diet induces an increase in testosterone and thus contributes to an increase in blood pressure by reducing the levels of *Mycobacterium avium* and its metabolite arachidonic acid (Vaziri et al., 2013). Bile acids exert antihypertensive effects through vasodilatory and anti-inflammatory mechanisms (Vítek and Haluzík, 2016): (1) bile acids activate Farnesoid X receptor (FXR) and TGR5 with vasodilatory activity, and supplementation with bile acids and their receptor agonists reduces blood pressure in animal models (Shi et al., 2021; O'Donnell et al., 2023); (2) bile acids act on dendritic cells (DCs) to promote the production of regulatory T cells (Tregs), inhibit inflammatory vesicle activation and inflammatory cytokine secretion, and develop an anti-inflammatory phenotype (Campbell et al., 2020). An increase in serotonin (5-HT) in the intestinal and somatic circulation inhibits afferent feedback from the vagus nerve to the cerebral nerves, thus promoting hypertension (Zubcevic et al., 2019). The presence of large numbers of sulfate-reducing bacteria in the mammalian colon results in the production of large amounts of sulfur-containing compounds, including hydrogen sulfide (H2S) (Lenka et al., 2016). H2S mediates vasodilatory effects by facilitating the opening of ATP-sensitive potassium channels, causing a decrease in blood pressure.

## 4 Intestinal microbiota and metabolic diseases in pregnancy

**Figure 3:**
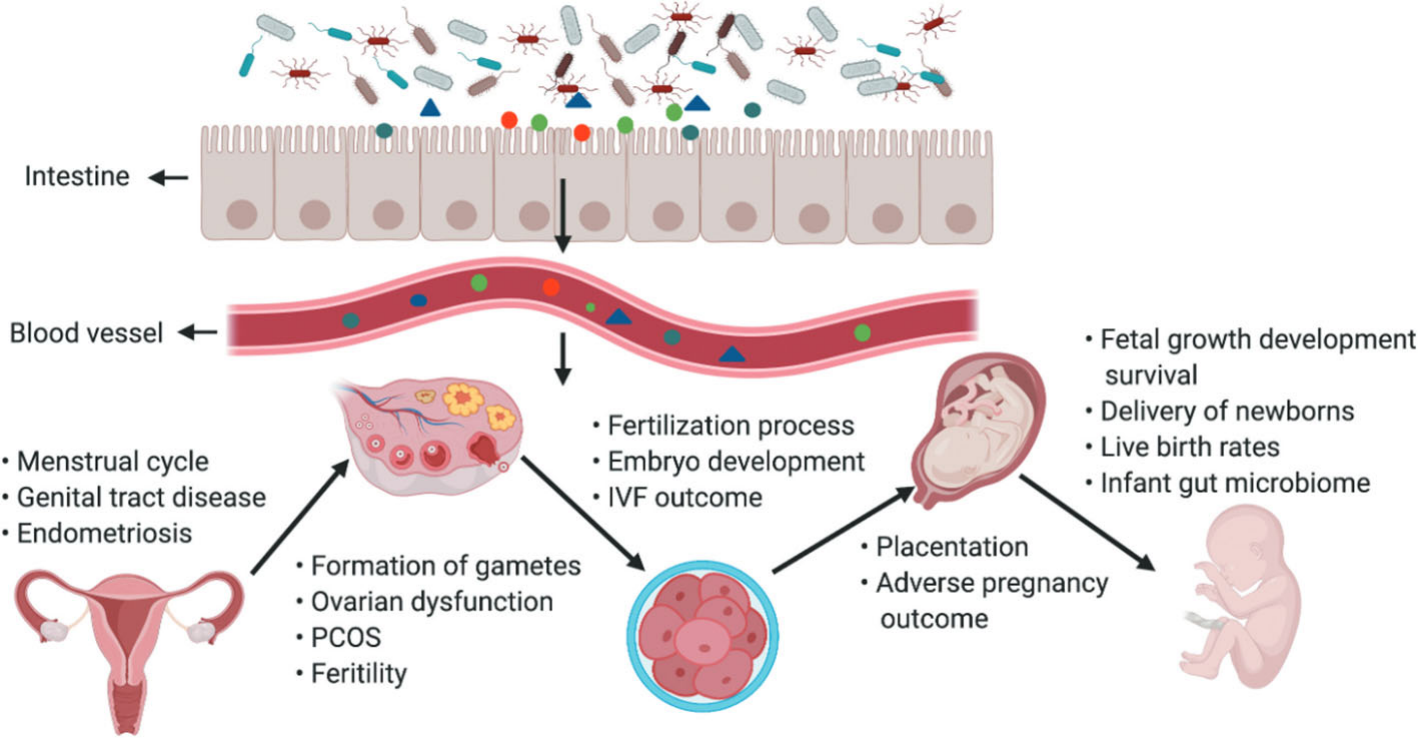
The gut microbiota and its impact on the female reproductive tract, embryo development and pregnancy. The gut microbiota and its impact on the female reproductive tract, embryo development and pregnancy. Products of gut microbiota may be transported through the circulation and influence the female reproductive tract, ovarian function, embryonic development and the health of the mother and fetus. The gut can affect the formation of gametes, embryo development and fertilization processes, and alteration of intestinal flora can lead to ovarian dysfunction, PCOS, infertility, and adverse IVF outcomes (Qi et al., 2021).

As a special group of pregnant women, it is crucial to study their intestinal microbiota (Ishimwe et al., 2021). To ensure greater energy intake to supply nutrients for their development and that of the fetus, pregnant woman’s body experiences an increase in adiposity and a decrease in insulin sensitivity, which is associated with self-regulation of the gut flora (Qi et al., 2021) (**Figure 3**). From early to late pregnancy, the diversity of the gut flora decreased, the abundance of the phyla Ascomycota and Actinobacteria increased, and the composition of the gut flora of pregnant women in early pregnancy was similar to that of nonpregnant women (Koren et al., 2012). When pregnant women are affected by obesity, GDM and HDP, and other metabolic diseases during pregnancy, their gut flora will undergo obvious changes (Asemi et al., 2012; Shepherd et al., 2017). The gut flora is a risk factor for obesity and dyslipidemia, with Eggerthella elevating triacylglycerol and lowering HDL cholesterol and Pasteurellaceae lowering triacylglycerol, suggesting that the gut flora plays an essential role in lipid metabolism (Fu et al., 2015). The number of bacteria in the intestinal tract of pregnant women, especially the number of Staphylococcus spp., is positively correlated with the total plasma cholesterol concentration and an increase in the number of Mycobacterium spp (Santacruz et al., 2010). Possible mechanisms by which the gut flora influences lipid metabolism include SCFAs produced by intestinal bacteria, which are substances that regulate intestinal immune homeostasis, influence peripheral metabolic tissues for energy expenditure and insulin sensitivity, and regulate lipid metabolism (Poll et al., 2021). Dietary phosphatidylcholine is metabolized by the gut flora to produce TMAO, which affects cholesterol and sterol metabolism through reverse cholesterol transport and directly affects blood lipid levels (Webster et al., 2019).

## 5 Intestinal microbiota and HDP

To ensure the healthy development of the mother and fetus and to effectively prevent and reduce adverse pregnancy outcomes for mothers and infants during pregnancy (Halkjaer et al., 2016). HDP is a common clinical pregnancy-specific acute and critical condition and is one of the leading causes of increased maternal and perinatal morbidity and mortality (Wertaschnigg et al., 2019). In the near term, HDP can lead to poor maternal and fetal outcomes, and in the long term, it affects the gut flora and long-term health of the offspring, as evidenced by a significantly increased risk of obesity and metabolic syndrome in the offspring (Hunt et al., 1994). There is a lack of effective preventive and therapeutic measures for HDP (Hajianfar et al., 2018). A relationship between intestinal microecological dysbiosis and HDP has been reported (Amarasekara et al., 2015). The species and abundance of the gut flora in pregnant women with preeclampsia were significantly altered compared to those in healthy pregnant women, with an increase in the abundance of pathogenic bacteria such as *Clostridium perfringens* and Bulleidia moorei and a decrease in the abundance of probiotic bacteria such as Coprococcus catus (Liu et al., 2017). The 16S rRNA genes of *Bacillus cereus*, Listeria, Salmonella spp., and Escherichia spp. were detected in the placental tissues of patients with preeclampsia after cesarean section compared with those of normal pregnancies and cesarean sections, suggesting that the gut flora is involved in the development of preeclampsia (Chen et al., 2020).

### 5.1 Characterization of the gut flora in patients with HDP

The gut flora of patients with PE is characterized by reduced secretion of SCFAs and, consequently, a reduced ability to produce beneficial bacteria, disrupting the dynamic balance of the gut flora and increasing pathogenic bacteria (Brantster et al., 2011). Clostridium belongs to the genus of gram-positive anaerobes that secrete toxins involved in arterial vasospasm and release antihypertensive substances that lead to an increase in blood pressure (Pyne et al., 2014). Prevotella is a genus of gram-negative anaerobes that expresses some reductase-like substances associated with oxidative stress and modulates inflammatory substances, accelerating immune disorders and infections (Tett et al., 2018). Fusobacterium is the most dominant genus of the phylum Clostridium. These gram-negative anaerobes are symbiotic in humans and animals and can accumulate in the colon, where they can cause bacteremia and various rapidly progressing infections (Brennan and Garrett, 2019). Fusobacterium is much more virulent and produces important lipopolysaccharides (LPS), endotoxins, and hemolysins, which increase its virulence (Submucosal microbiome of peri-implant sites: a cross-sectional study, 2021). Bifidobacterium is a gram-positive anaerobic bacillus that synthesizes vitamins and functions with other anaerobes to form a biological barrier on mucosal surfaces, preventing exogenous bacteria from attacking the body, stimulating immune function, activating macrophages, and improving host resistance to infection (Miao et al., 2021). Fecalibacterium is expressed at a high level in healthy human subjects, reduces adipose and intestinal tissue inflammatory expression, maintains intestinal integrity, and ameliorates inflammatory damage (González-Sarrías et al., 2018).

### 5.2 Gut flora influence HDP mechanisms

**Figure 4:**
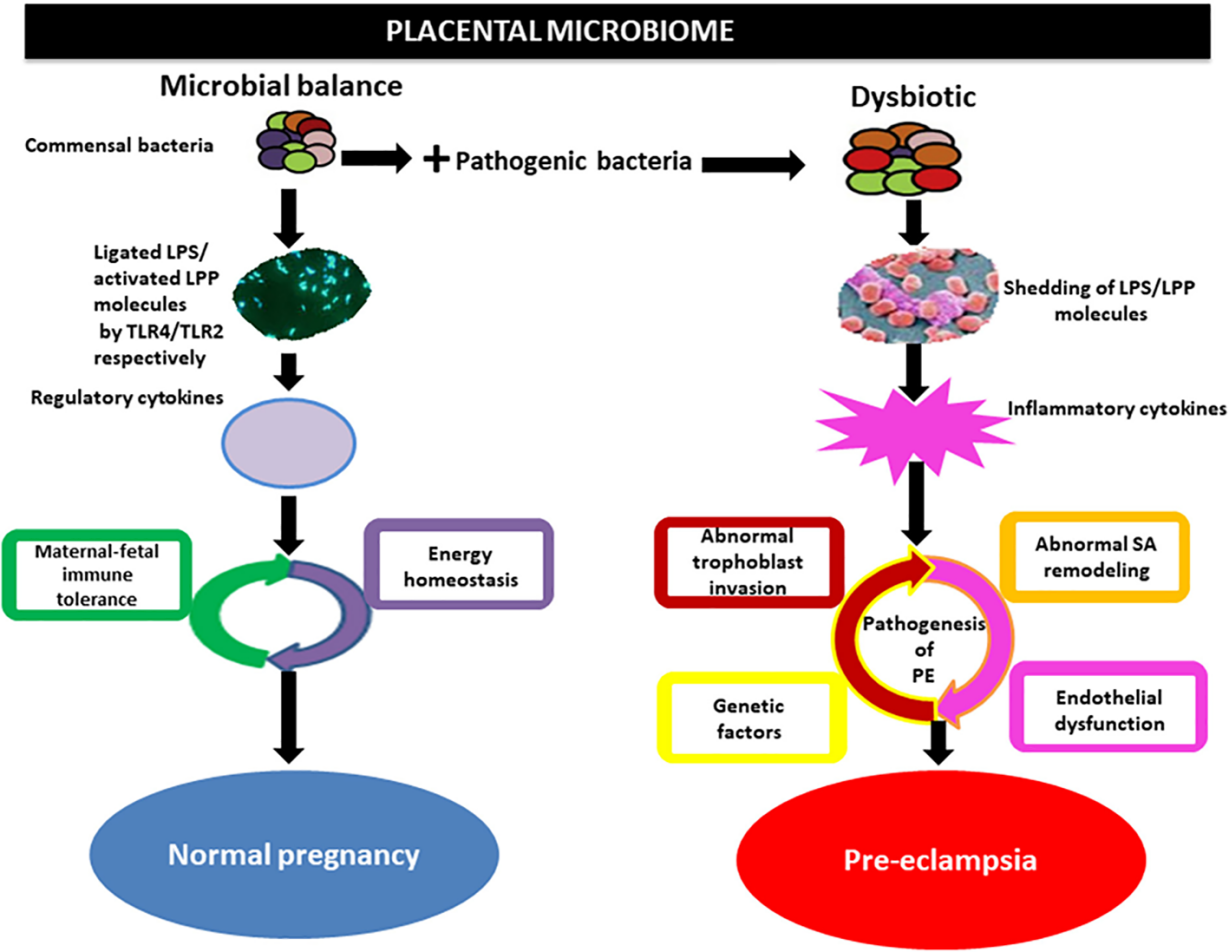
Implication of the placental microbiome in preeclampsia. Alterations in placental structures could result from the direct action of pathogenic bacteria through the release of LPS or lipoproteins (LPPs), which activate inflammatory cytokines via interactions with Toll-like receptors (TLRs), especially TLR4/TLR2. This invokes the pathogenic process of preeclampsia. Under physiological conditions, commensal bacteria release bacterial products such as LPS and LPP from gram-negative and gram-positive bacteria, respectively, which are ligated by TLR4 and activated by TLR2 located on the surface of trophoblasts. This in turn activates regulatory cytokines to promote a tolerogenic microenvironment and maintain energy homeostasis in normal pregnancy. SA, spiral artery Olaniyi et al., 2020().

The gut flora can affect blood pressure by influencing the production of hormones such as 5-HT, dopamine, and norepinephrine (Lyte, 2014). Systemic inflammatory immune overreaction and inflammation-mediated vascular endothelial cell injury are the pathological mechanisms of HDP (American College of Obstetricians and Gynecologists'Committee on Practice Bulletins-Obstetrics, 2019). The mechanism by which multisystemic injury occurs in patients with HDP is due to a series of inflammatory factors produced by the placenta that cause oxidative stress to placental tissues, which leads to endothelial injury of the maternal vasculature, resulting in multisystemic pathology and the development of symptoms such as hypertensive disorders and proteinuria (Verhaar et al., 2020). Inflammatory factors and intestinal microecological imbalance, as well as bacterial translocation, mutually promote and exacerbate the inflammatory response of the organism via mechanisms such as an imbalance of the gut flora, an increase in the proportion of conditionally pathogenic bacteria, and a decrease in the proportion of commensal bacteria (Johnson et al., 2020). In particular, a decrease in the number of bacteria that protect the intestinal barrier leads to an increase in intestinal permeability, which allows endotoxins, such as LPS, to enter the body and be recognized by immune cells to produce a variety of inflammatory factors, which can cause extensive damage to the organism (Hsieh et al., 2020). There is mutual regulation and mutual reinforcement in the HDP-inflammatory response-gut flora imbalance (Lamichhane et al., 2021). Certain bacteria and metabolites in the gut flora affect blood pressure levels in pregnant women, suggesting that these flora and their products can be used as characteristic screening markers for HDP disease risk and that monitoring these specific flora can be used as indicators of the efficacy of HDP treatment and prognosis (Olaniyi et al., 2020) (**Figure 4**).

Changes in the gut flora of pregnant women during normal pregnancy are due to their involvement in numerous physiological activities, such as maternal nutrition, metabolism, and immunity (Ekaterina et al., 2014; Su et al., 2016). There are corresponding cyclic changes in maternal gut flora during pregnancy (Faas et al., 2020). During pregnancy, as gestation progresses, the physiological gut flora changes, with a decrease in cellular diversity and an increase in the abundance of pathogenic bacteria (Lv et al., 2019). The abundance of pathogenic bacteria such as Clostridium, Fournierella, Prevotella, and Fusobacterium increased, and the abundance of Bifidobacterium, Fecalibacterium and other beneficial bacteria decreased3rd International Genomic Medicine Conference (3rd IGMC 2015), 2016. This is related to immune system alterations during pregnancy, where the gut flora influences immune cell activation, leading to an increase in pathogenic bacteria and accelerating the development of intestinal diseases. In the pathological state of HDP, changes in the gut flora caused by a normal pregnancy state are superimposed on the pathological alterations in the gut flora caused by hypertensive disorders, making the relationship even more complex (Sun et al., 2020). Compared with the gut flora of healthy pregnant women at 26/36 weeks, there were differences in the gut flora of patients with PE at 26/36 weeks, which were mainly characterized by an increase in the abundance of pathogenic bacteria and a decrease in the abundance of beneficial bacteria (Chang et al., 2020). The reason for this is that norepinephrine and sympathetic nerve activity are increased when blood pressure is elevated, leading to increased intestinal permeability and an increased intestinal inflammatory response, which further disrupts the gut flora, and pathogenic bacterial activity increases and intensifies with the duration of the disease (Karbach et al., 2016; Santisteban et al., 2016). The association between PE and infection of the gut flora is closely related to the duration of pregnancy, so clinicians should focus on the gut flora of pregnant women with HDP in the late stages of pregnancy. Screening can reduce the adverse effects on fetal development (Sun and Niu, 2020)

### 5.3 Maternal intestinal microecology affects the establishment of intestinal microecology in offspring

The maternal microbiota is an essential source of gut flora in offspring, and the infant’s gut flora is derived from the mode of delivery, feeding practices, antibiotics, and environmental exposures (Sauer and Grabrucker, 1295). Compared to those of newborns delivered by cesarean section, the gut flora of normal newborns overlap with the maternal vaginal flora community (Dominguez-Bello et al., 2010). Simultaneous sampling of the vagina, skin, breast milk, oral cavity, and feces of infants and their mothers up to 4 months postpartum revealed that maternal vaginal and skin flora could reside temporarily in the infant’s gut and then gradually be replaced by more persistent intestinal microbiota as the infant develops (Ferretti et al., 2018). During pregnancy, nutrients are transported to the fetus via the placenta, which impacts the establishment of fetal gut flora, and during breastfeeding, the infant’s intestinal microbiota can be altered through the breastfeeding route (Chu et al., 2016). Antibiotic use during pregnancy interferes with the gut flora of pregnant women, whereas scientific supplementation with probiotics and prebiotics may positively impact the mother and her offspring (Nishitsuji et al., 2017). A 3-month follow-up analysis of probiotic supplementation during pregnancy and postpartum in pregnant women with a history of antibiotic treatment revealed that breastfed infants had an increase in bifidobacteria and a decrease in Aspergillus and *Clostridium difficile* in their intestinal microbiota, reducing or even eliminating the effects of antibiotics (Korpela et al., 2018).

### 5.4 Intervention strategies for HDP targeting the intestinal microbiota

The selective intervention of the intestinal microbiota provides new ideas for the prevention and treatment of HDP (Callaway et al., 2016b; Taddei et al., 2018). By intervening in the intestinal microecological balance, the inflammatory state and immunomodulatory function of pregnant women can be improved (Manzanares et al., 2016). The intestinal microbiota distribution in preeclampsia patients showed a sharp decrease in the abundance of SCFAs and their producer bacterium Mucinophilus-Eckermannia (Jin et al., 2022). A high-fat, high-protein diet in hypertensive patients increases the abundance of harmful bacteria in the intestinal tract, leading to intestinal microbiota dysbiosis, and harmful bacteria can increase blood pressure by promoting vasoconstriction (Turnbaugh et al., 2006). Bifidobacterium can convert cholesterol into steroids, lowering lipid concentrations and thus lowering blood pressure. When the number of bifidobacteria decreases, the flora becomes dysbiotic, inducing the release of renin and increased blood pressure (Kalliom Ki et al., 2008; Melania et al., 2010). Interventions for intestinal microecology include dietary modifications, probiotic supplementation, and fecal transplants (Everard et al., 2011; Durgan et al., 2016; Gallo et al., 2016).

#### 5.4.1 Dietary modifications

Dietary habits play an essential role in the composition of the gut flora (David et al., 2014). Low-fat, high-fiber diets, and other dietary interventions have been associated with lower blood pressure; low-fat diets such as yogurt and fruits are recommended to reduce hypertension, effectively reducing the incidence of HDP (Estruch et al., 2013). A strict vegetarian diet in normal subjects was found to reduce the abundance of pathogenic bacteria such as Enterobacteriaceae and increase the abundance of *Bacteroides fragilis* and Clostridium spp., thereby reducing the SCFA concentration (Nutting et al., 1991). A high-fiber, low-fat diet increases gut flora abundance and improves obesity in normal subjects (Marques et al., 2018). Animal studies have shown that *Clostridium difficile* expression is increased in mice with low dietary fiber and promotes the development of hypertension and cardiac remodeling (Kaye et al., 2020). A meta-analysis conducted by REYNOLDS et al., which included hypertensive patients, demonstrated that dietary fiber led to a reduction in systolic blood pressure, with an additional intake of 5 g of dietary fiber per day resulting in a decrease in systolic blood pressure of 2.8 mm Hg and diastolic blood pressure of 2.1 mm Hg (Reynolds et al., 2022).

#### 5.4.2 Probiotic supplementation

Probiotic therapy targeting the gut flora significantly impacts HDP intervention (Hsu et al., 2022). Probiotics exert antihypertensive, lipid-lowering, and anti-inflammatory effects by producing antihypertensive bioactive peptides, modulating gut flora metabolites, and mediating other pathways, such as sympathetic nerves, nitric oxide, and inflammation (Kobyliak et al., 2015; Gómez uzmán et al., 2016). Synergism, a microbial blend of probiotics and prebiotics, targets the intestinal microbiota. A meta-analysis incorporating 11 randomized controlled studies reported that synbiotic intervention significantly reduced systolic blood pressure levels in adults after 12 weeks of intervention (Hadi et al., 2021). CB-GLP-1 can maintain intestinal homeostasis by upregulating the abundance of Lactobacillus spp (Lafferty et al., 2018). On the other hand, CB-GLP-1 mediates the initiation of blood pressure lowering mechanisms by the renin-angiotensin-aldosterone system via glucagon-like peptide-1 and butyric acid (Yin et al., 2020; Wang et al., 2023). Probiotic-fermented milk is effective at lowering blood pressure in hypertensive and prehypertensive patients, probably because lactobacilli and bifidobacteria can control blood pressure by breaking down proteins to produce peptides that inhibit angiotensin-converting enzyme activity, and the intestinal microbiota can control blood pressure by breaking down food to produce substances with estrogen-like effects (Dong et al., 2013). Probiotic preparations, such as Bifidobacterium bifidum and *Lactobacillus acidophilus*, given to pregnant women in mid-pregnancy can improve the immune status and inflammation level of pregnant women and reduce the severity of hypertensive disorders during pregnancy (Robles-Vera et al., 2020a).

#### 5.4.3 Fecal bacteria transplantation

FMT is a method of isolating healthy human gut flora and transplanting it into a patient’s intestine to cure the disease by reestablishing the patient’s gut flora (Smits et al., 2018). A study on nonpregnant women revealed that insulin sensitivity improved after duodenal instillation of nonmetabolically syndromic gut flora in patients with metabolic syndrome. FMT has been used to successfully alleviate intestinal infections and weight loss in several obese patients by altering the structure and metabolism of their gut flora (Vrieze et al., 2012). Due to the complexity of the gut flora, the long-term efficacy and safety of FMT are still unclear (Young, 2019). The effects on the mother and child are even more unclear, with no reports of fecal transplants during pregnancy. Many prospective and interventional studies are needed to explore the specific flora and mechanisms of action involved (Khoruts and Weingarden, 2014; Mell et al., 2015).

### 5.5 Clinical value and challenges of gut flora testing applied to HDP

Monitoring the intestinal microecological balance is essential for understanding the mechanisms of HDP and for the prevention and treatment of HDP (Hsu et al., 2018). Due to the special characteristics of pregnant women’s physical and metabolic conditions, the disease’s pathological process superimposed on the transformation of the intestinal microbiota is more complex (Mokkala et al., 2020). For the sequencing of the intestinal microbiota before laboratory testing, the following technical difficulties need to be overcome: (1) the testing database of the intestinal microbiota must have sufficient strain coverage and depth; (2) the quantification method of the intestinal microbiota needs to have high sensitivity, and the calculation method needs to have high precision and less error; (3) the differences in flora caused by the heterogeneity of the individual patient need to be carefully differentiated; and (4) most of the studies are still in preclinical research and cannot fully translate rodent research to the relevant physiology and pathology of the human body (Chen et al., 2019). Some scholars have taken into account the heterogeneity of host physiology and immune responsiveness and have combined the specificity of pregnancy physiology itself and the changes in the abundance of the combined flora of inflammatory indicators and lipid metabolism indicators related to the pathological process as a means of comprehensively evaluating host immune tolerance to the intestinal microbiota, which is very important for the prediction of the risk of HDP and precise intervention (Houttu et al., 2017).

## 6 Conclusion

The abundance of the intestinal microbiota in patients with HDP decreases, and the structure of the bacterial community is significantly altered, as manifested by a decrease in beneficial bacterial genera and an increase in pathogenic bacterial genera. An imbalanced intestinal microbiota leads to intestinal barrier dysfunction, causing persistent systemic inflammation that promotes the occurrence and development of HDP. There is still a need for further research on the relationship between HDP and immune regulation of the intestinal microbiota, the role of the mechanism underlying the HDP-inflammatory response and intestinal microbiota imbalance and the relationship between the HDP-immune response and intestinal microbiota imbalance. With the implementation of precision medicine and extensive health management, as well as the broad application of high-throughput technologies such as macrogenomics and metabolomics, the relationship between intestinal microecology and HDP will be further revealed, and the study of the exact cellular and molecular levels of the mechanism will be a hot topic in the future. The evaluation of drug safety, the assessment of efficacy, and the screening of euglycemic effects from the level of intestinal microecology will be the direction of clinical research. Comprehensive optimization of the existing clinical medical strategies during pregnancy and childbirth will have a long-term impact on the health of mothers, infants, and children with HDPs, so the role of intestinal microbiota testing in the future diagnosis and treatment of HDPs will become increasingly extensive, and the value of clinical application will become increasingly reasonable.
